# Predicting the Development of Adult Nature Connection Through Nature Activities: Developing the Evaluating Nature Activities for Connection Tool

**DOI:** 10.3389/fpsyg.2021.618283

**Published:** 2021-03-23

**Authors:** Victoria Carr, Joelene Hughes

**Affiliations:** RSPB Centre for Conservation Science, The Lodge, Sandy, United Kingdom

**Keywords:** connection to nature, nature connection, nature relatedness, nature activities, evaluation, pro-environmental behavior, conservation behavior

## Abstract

Nature Connection (NC) is considered an important driver of conservation behavior. Consequently, conservation organizations run many activities aiming to increase NC among participants. However, little is known about which activities are most effective at doing this and why. This study developed the Evaluating Nature Activities for Connection Tool (ENACT), to evaluate the effectiveness of activities for increasing participants’ NC and nature-related intentions. ENACT comprises 11 activity aspects identified through two research phases. In Phase 1, a literature search, focus group and interviews identified desired, short-term behavioral outcomes of nature activities, and variables that might promote these. In Phase 2, 241 adults completed a pilot survey immediately post-nature activity, with 1-month follow-up (*N* = 145), to evaluate the impact of participation on NC, nature-related behavioral intentions and behaviors. ENACT correlated with NC measures and offered incremental validity in predicting nature-related behavioral intentions and self-reported behaviors after 1 month.

## Introduction

Nature is declining globally at an unprecedented rate, with around 1 million animal and plant species threatened with extinction (Intergovernmental Science-Policy Platform on Biodiversity and Ecosystem Services, 2019). Although species and habitats are in decline in the United Kingdom (Hayhow et al., 2019), a 2017 survey showed that 47% of the United Kingdom population was unaware or unconcerned about biodiversity loss, with a further 42% showing only “some engagement” (Department for Environment, Food and Rural Affairs, 2019). If people do not value nature or see it as relevant to their lives, they will likely remain unconcerned about its loss and be less likely to invest in its protection (Miller, 2005; Swaisgood and Sheppard, 2011; Soga and Gaston, 2016). The conservation community recognizes that successful biodiversity conservation requires empowering more people to act positively for nature; the challenge lies in identifying effective interventions and activities to achieve this. Thus, a critical area for theoretical and applied research is to understand how nature activities promote conservation behavior.

Conservation behavior encompasses any activity that supports sustainability by reducing harmful behaviors or adopting helpful ones (Saunders, 2003; Clayton, 2012). Changing human behavior is a crucial route to conservation success (Schultz, 2011). Solely providing information about an issue, the knowledge-deficit model, does not successfully change behavior nor implement conservation solutions (McKenzie-Mohr, 2011; Toomey et al., 2017). Nature Connection (NC; or Connection to Nature) has emerged as a key factor promoting conservation behavior (Zylstra et al., 2014; Mackay and Schmitt, 2019; Whitburn et al., 2019; Martin et al., 2020). Consequently, increasing NC within individuals and populations is considered important practice for conservation organizations that seek to promote conservation behavior, and NC provides the theoretical basis for many activities that take place on nature reserves. This research aims to determine the aspects of nature activities (both aspects that directly promote NC and those that indirectly support NC) that predict conservation behavior, and provide a suitable tool to evaluate them.

Nature connection is a broad psychological construct describing an individual’s enduring relationship with nature and their perception of belonging to a wider natural community (Mayer et al., 2009; Cheng and Monroe, 2012; Zylstra et al., 2014), including identity, affective, cognitive, and behavioral elements (Kals et al., 1999; Schultz, 2002; Mayer and Frantz, 2004; Olivos and Aragonés, 2011; Kals and Müller, 2012; Tam, 2013; Hatty et al., 2020). Long-term (or trait) NC has been linked to numerous outcomes, including conservation behavior and human health and wellbeing (Gosling and Williams, 2010; Capaldi et al., 2014; Frantz and Mayer, 2014; Mackay and Schmitt, 2019; Whitburn et al., 2019; Martin et al., 2020). Although NC research has built rapidly, major questions remain about the pathways through which NC develops and influences different outcomes (Zylstra et al., 2014; Cleary et al., 2018). For example, which strategies or interventions are most effective for cultivating NC and why?

A long-term, stable NC is unlikely to arise from a single event (Nisbet and Zelenski, 2013; Lumber et al., 2017). Developing NC is likely to require repeated activities that each increase short-term (state) NC whilst combining over time to build the long-term (trait) NC that will influence people’s long-term behavior (Schultz and Tabanico, 2007; Whitburn et al., 2019; Hatty et al., 2020; see hypothesized pathway in Supplementary Material 1). However, conservation organizations can often only provide single-event nature activities. In order to use limited resources to greatest effect, they need a better understanding of how to develop and evaluate state NC through single-event activities, so that they can help to build trait NC and conservation behavior over time.

While NC research has identified some example activities that may increase NC (e.g., Beery, 2013), there is scant literature on which aspects of activities promote NC. A notable exception is Lumber et al. (2017), who examined relationships between NC and nine possible ways of engaging with nature, based on the Biophilia hypothesis (Kellert and Wilson, 1995). Lumber et al. identified five pathways for improving NC that can potentially be incorporated into activities: Contact (engaging with nature through the senses); Beauty (perception of the esthetic qualities of nature); Emotion (affective state or sensation that occurs as a result of engaging with nature); Meaning (using natural symbolism to communicate a concept); and Compassion (extending the self to include nature, leading to concern for other natural entities). Other activity aspects proposed to influence development of NC include awe and wonder (Perkins, 2010; Yang et al., 2018), sense of place (Masterson et al., 2017), empathy and sympathy (Cheng and Monroe, 2012), and self-reflection (Richardson and Sheffield, 2015).

Zylstra et al. (2014) pointed out a shortage of research evaluating the effectiveness of interventions that aim to increase NC, and this situation continues. A key barrier to evaluating the success of NC activities is the lack of appropriate instruments for assessing change in NC over shorter timeframes. Currently there are at least 14 published NC measures (Hatty et al., 2020), including the Connectedness to Nature Scale (CNS; Mayer and Frantz, 2004), Nature Relatedness scale (NR and short-form NR-6; Nisbet et al., 2009; Nisbet and Zelenski, 2013), Environmental Identity scale (Clayton, 2003), and Nature Connection Index (Richardson et al., 2019). Almost all existing measures assess trait NC and are therefore unsuitable for evaluating the effectiveness of single-event activities. For example, it is unrealistic to expect meaningful change in responses to items such as “I always think about how my actions affect the environment” (NR-6; Nisbet and Zelenski, 2013) after a nature experience lasting a matter of hours. Indeed, scores on Hatty et al.’s (2020) CN-12 measure remained stable over 12 months. Mayer et al. (2009) created a state version of the CNS to measure short-term fluctuations in NC, by altering wording to make items more specific to current feelings. Whilst the state CNS is more relevant to evaluating activities, it is relatively long (13 items), with complex language that can appear flowery to respondents (e.g., “At the moment, I am feeling embedded in the broader natural world, like a tree in a forest”).

There are further critical barriers to using existing NC measures to evaluate the effectiveness of nature activities. NC measures assess an individual’s level of NC (either trait or state) but do not provide direct feedback on an activity itself or information about why an activity has affected NC. Therefore a NC measure in isolation cannot provide practitioners with the necessary understanding about what makes some activities more effective than others and how activity effectiveness can be improved. In addition, existing NC measures were not designed to predict specific outcomes; for example, they are not designed to target just those aspects of NC that best predict conservation behavior, which is a key goal in this context.

Attributing behavior change to participation in a single activity is very difficult. However, it may be possible to evaluate an activity’s effectiveness in promoting conservation behavior by assessing the short-term affective and cognitive responses elicited by the activity that predict short-term outcomes thought to link to future behaviors (see Supplementary Material 1). For example, by measuring thoughts and feelings arising from partaking in a single event, and assessing how these link to short-term outcomes, such as nature-related behavior intentions and short-term behaviors. This is consistent with the Theory of Planned Behavior (Ajzen, 1991), whereby attitudes, norms, and perceived control shape behavioral intention, which in turn predicts behavior. Although the well-documented intention-behavior gap (e.g., Kaiser et al., 1999; Sheeran and Webb, 2016) shows that intention does not necessarily translate into behavior, conservation behavioral intention has been shown to be a key predictor of conservation behavior (Bamberg and Möser, 2007; Mackay and Schmitt, 2019). Intention may be particularly relevant in the context of evaluating short-term activities, where there is limited opportunity to demonstrate actual behavior. Thus, a tool to specifically assess the aspects of activities and state NC that are relevant to nature-related behavior intentions and short-term behaviors would be a crucial development.

This study aimed to develop a short, practical survey tool (ENACT) that could be used to evaluate the effectiveness of nature activities that are designed to connect adults to nature in order to promote future conservation behavior. Underpinned by NC theory, the goal was to determine the aspects of nature activities – both those directly related to NC, and other supportive features (e.g., facilitation) – that best predict short-term outcomes (e.g., intentions), and use these to develop an evaluative tool for single-event activities (see Supplementary Material 1). We focused on adults rather than children, as adults have great agency for conservation behaviors within a much shorter timeframe, yet to date attention has focused primarily on developing children’s NC (Cleary et al., 2018). As an applied tool, it was essential that ENACT should be short, straightforward, easy to use and provide practical information to enable practitioners to improve their nature activities.

We used a two-phase, mixed methods approach to develop ENACT. In Phase 1, we used multiple methods to identify (a) the desired short-term outcomes from NC activities seeking to promote conservation behavior, and (b) a range of activity aspects that may influence these outcomes, from the perspective of research, practitioners and participants. In Phase 2, we used the results from Phase 1 in a pilot survey to identify the activity aspects that best predicted the desired outcomes, with results refining the survey into a short evaluative tool (ENACT).

## Phase 1: (A) Identifying Nature Activity Outcomes and (B) Developing Enact Items to Assess Activity Aspects

### Ethics Statement

Ethical approval for both Phases of the research was gained from the RSPB Centre for Conservation Science Human Ethics Committee. All participants provided informed, written consent, and were debriefed. Participation was voluntary with no incentive or reward.

### Materials and Methods

This study was conducted at the RSPB, the United Kingdom’s largest nature conservation charity. The RSPB runs a large number of nature reserves that conduct people engagement activities across a wide variety of environments. We made the assumption, supported by information from internet searches, that the RSPB offers similar nature activities and faces similar challenges to other organizations in the United Kingdom nature conservation sector. We combined the following three sources of information to determine (a) short-term outcomes relevant to conservation behavior that could realistically result from engagement in a single nature activity, and (b) aspects of nature activities that may drive these short-term outcomes.

#### Literature Search

To gain an informed picture of current scientific evidence on the potential aspects of nature activities that may influence the development of NC in adults, we conducted a broad review of recent peer-reviewed and gray literature. We identified papers and reports from searches in electronic databases: Google Scholar, PsychSource, and EBSCO Discovery Service (using terms “Connection to nature” NOT children from 2010 onward); information from RSPB and academic experts, plus citations in and of the Zylstra et al. (2014) review.

#### Stakeholder Focus Group

Twelve RSPB employees (67% female, 33% male) with responsibility and/or expertise in NC took part in a 2.5 h focus group that we facilitated. The discussion focused on four questions that targeted different research purposes:

Question 1. Which RSPB experiences might help to develop connection in adults? (To identify the range of relevant activities for evaluation)
Question 2. How do adults develop connection to nature & how can the RSPB influence this? (To develop a hypothesized adult connection pathway)
Question 3. What connection outcomes do we want to achieve through RSPB experiences? (To identify desirable, short-term outcomes indicating a successful connection activity)
Question 4. What aspects of RSPB experiences help to increase connection? (To identify activity aspects that may promote NC and short-term outcomes)

#### Interviews With Nature Activity Participants

We conducted semi-structured, qualitative interviews with 30 adult members of the public who had just attended a RSPB nature activity (53% female, 47% male; mean age 55 years, range 19–78 years). One author (VC) conducted the interviews after three different nature activities, held at three nature reserves in England. The interview lasted around 10 min and asked open-ended questions covering: participants’ feelings and thoughts after the nature activity, which aspects of the activity stimulated these, whether the activity enhanced participants’ sense of connection to nature and how they described this, and behaviors that participants intended to do after the activity (see Supplementary Material 2). All adults leaving each activity after at least 1 h were approached to participate (as far as feasible). We audio-recorded and content analyzed the interviews (e.g., Krippendorff, 2004) to identify activity factors, outcomes, and participant wording used.

### Results

#### Nature Activity Outcomes (a)

The focus group identified a wide range of existing nature activities that may promote adult NC (Question 1), and supported the hypothesis that repeated activities are necessary to develop enduring NC and changes in conservation behavior (Question 2). Most nature activities aimed to foster NC and nature-related behavior generally, rather than targeting specific conservation behaviors or actions (Question 3). It was therefore not feasible to identify specific conservation behavior outcomes (as advocated by McKenzie-Mohr, 2011) that could be attributable to participation in a single activity yet applicable across a wide range of different activities. From the focus group, the key desired activity outcome was that participants ended the activity with the intention to take, and subsequently did take, steps to repeat or reinforce their nature experience, thus creating further opportunities to build NC and conservation behavior (in line with our hypothesized pathway in Supplementary Material 1).

Interview participants had spent an average of 2 h at the reserve; 20% said it was their first time at a RSPB nature reserve, whereas 33% visited RSPB reserves at least monthly. In addition to revealing a variety of feelings and thoughts post-activity [see results in section “Nature Activity Aspects (b)”], interview participants identified a number of behaviors they might intend to do following a nature activity. The most frequently cited behaviors were: returning to the same nature reserve (mentioned in 83% of interviews), telling others about the activity (47%), and visiting another nature reserve (33%; Supplementary Material 3).

We triangulated data from the focus group and interviews to identify a range of short-term behaviors that represented successful outcomes for a nature activity and used these to create an 8-item index of desirable, nature-related behavioral intentions (BI; Supplementary Material 4). The eight items comprised three sub-themes: intentions to repeat nature activities (e.g., I intend to visit this nature reserve again); intentions to share experiences with others, which reinforces the positive experience (e.g., I intend to tell other people about today’s event), or intentions to follow up with action or information-seeking (e.g., I intend to take action to help nature). The response scale was framed as whether participants intended to do each behavior within the next month and rated on a five-point Likert-style rating scale (from 1 = “Definitely not” to 5 = “Definitely”), similar to that used by Harland et al. (1999). Five-point rating scales have been found to yield higher quality data than seven or 11-point scales and to be preferable for use with the general public (Weijters et al., 2010; Revilla et al., 2014; Robinson, 2018). BI scores were calculated as the mean of all eight items, in line with recommended practice (Robinson, 2018), and were only calculated for participants who responded to all eight items. For the pilot survey only, we added one qualitative free-text item, asking participants who intended to take action to help nature what action(s) they intended to take.

#### Nature Activity Aspects (b)

Interview participants provided a wide range of responses regarding their feelings and thoughts after the nature activity, which aspects of the activity they felt stimulated these and/or made them feel more connected to nature. The most frequent themes in the interviews were: seeing something new or special during the activity (mentioned in 83% of interviews), general enjoyment (73%), contact with nature (70%), quality of facilitators (63%), and feelings of excitement/awe (60%). Through triangulation of the outputs from the interviews, focus group (Question 4) and literature search, we identified a pool of over 50 activity aspects that could potentially influence NC and/or nature-related behavioral intention (see Supplementary Material 3). All of these aspects were mentioned in at least one interview; 33 of the aspects were also mentioned in the focus group; and 22 appeared in the literature search.

It was not feasible to test all of these aspects in the pilot survey, as it would have made the survey unacceptably long for respondents in an applied setting (Maloney et al., 2011; Hatty et al., 2020). We therefore prioritized the aspects for inclusion. We retained aspects mapping to the Lumber et al. (2017) five pathways due to their theoretical significance. Further aspects were included if they were mentioned in eight or more interviews (i.e., over 25%). We developed a set of 20 pilot survey items to cover the selected activity aspects (see Table 1); some items covered more than one related aspect (see Supplementary Material 3). Item wording needed to be suitably generic to apply to a wide range of different nature activities. Where appropriate, we used or adapted existing items from other surveys (e.g., Monitor of Engagement with the Natural Environment; Natural England, 2018). Where possible, we used verbatim wording from interview participants in item content to increase face validity and aid comprehension (DeVellis, 2017). We produced two items each for four of the Lumber et al. (2017) NC pathways (Contact, Beauty, Emotion, and Compassion); for Emotion, we used the two most frequently-mentioned specific emotions (Calm and Excitement). We could only develop one suitable item for Meaning, which also linked with “sense of place” frameworks (Masterson et al., 2017). The remaining 11 items covered: activity facilitation; activity organization; learning; novelty; interest; distraction from stress (similar to “clearing one’s thoughts”; Korpela et al., 2008); access to nature; immersion in nature; wildlife non-disturbance; awareness of conservation work and presence of others. The response scale was a 5-point Likert-style rating of extent (e.g., Clark and Watson, 1995), from 1 = “Not at all” to 5 = “A great deal.”

**TABLE 1:** Activity aspects item descriptives and correlation with behavioral intention (BI; *N* = 193).

Activity aspect	Item	Mean	SD	*r*_s_ with BI
Contact A	I got up close to nature	4.04	1.11	0.25
Contact B	I used different senses to experience nature (sight, sound, smell, touch)	3.59	1.26	0.35
Beauty A	I noticed beautiful things in nature	4.44	0.78	0.37
Beauty B	I took time to appreciate my surroundings	4.10	1.07	0.36
Emotion A	It made me feel calm and relaxed	4.19	0.91	0.45
Emotion B	It made me feel excited and amazed	3.52	1.18	0.47
Meaning	This place means something to me	3.89	1.19	0.35
Compassion A	It made me feel more responsible for protecting nature	3.53	1.23	0.35
Compassion B	It made me more concerned about the problems facing nature	3.08	1.25	0.24
Conservation awareness	It made me aware of the conservation work being done here	3.71	1.19	0.19
Facilitation	The staff/volunteers were knowledgeable	4.70	0.63	0.36
Immersion	I felt surrounded by nature	4.45	0.81	0.27
Interest	It was interesting and informative	4.27	1.03	0.38
Learning	I learned something new about nature	3.27	1.52	0.47
Nature access	I had privileged access to natural places	4.02	1.11	0.25
Non-disturbance	I was able to enjoy wildlife without disturbing it	3.99	1.15	0.25
Novelty	I saw wildlife/nature that I had never, or hardly ever, seen before	2.97	1.57	0.33
Organization	It was well organized	4.52	0.74	0.25
Presence of others	I saw that other people were interested in nature	4.14	0.94	0.26
Stress distraction	It took my mind off stresses or problems	3.92	1.19	0.46

## Phase 2: Pilot Survey and Enact Item Selection

### Materials and Methods

To determine the most appropriate items from Phase 1 for inclusion in ENACT, we conducted a quantitative pilot survey with adult activity participants at reserves. We invited participants to take part in a follow-up survey to validate the final tool.

#### Participants

Power analysis using the pwr package in R (Champely, 2018) indicated that a sample of 205 was needed for a multiple regression model using 20 predictors with a conservative estimate of *R*^2^ = 0.10 (*N* = 101 if *R*^2^ = 0.20). Tabachnick and Fidell (2001) and Field (2009) also suggest *N* = 200–210 for multiple regression with 20 predictors. Target sample size was *N* > 210 to allow for errors in completion. Participants were 241 adult members of the public who had just attended a RSPB nature activity (58% female, 40% male, and 2% undisclosed; mean age 51 years, range 19–87; and mean visit duration 2.7 h). 14% of participants said they had never visited a nature reserve before; 41% visited reserves once every few months or less, and 42% visited reserves at least monthly (2% blank). We removed a further eight participants during data checking as they endorsed the most positive response option for every activity aspect, suggesting possible response bias (e.g., Paulhus, 1991).

Two hundred and fourteen participants (89%) agreed to receive the follow-up survey, from which 145 follow up responses were received (68% response rate, 60% of full sample). Follow-up participants were 61% female, 38% male, and 1% undisclosed; mean age 52 years, range 19–74; mean visit duration 2.7 h; 14% had never visited a reserve before and 50% visited reserves at least monthly. Comparison of follow-up participants with participants lost to follow-up indicated little evidence of attrition bias. Although follow-up participants visited reserves more frequently than those lost to follow-up (*U* = 7711.5, *z* = 2.03, and *p* = 0.043), there were no significant differences between the two groups in terms of gender [*X*^2^(1, *N* = 237) = 1.13, *p* = 0.287], education level [*X*^2^(1, *N* = 230) = 0.12, *p* = 0.733], visit duration (*U* = 7185.0, *z* = 0.92, and *p* = 0.359), age [*t*(224) = −1.59, *p* = 0.114], trait NC [*t*(232) = −1.52, *p* = 0.129], state NC [*t*(231) = −1.18, *p* = 0.239], ENACT score [*t*(216) = −0.80, *p* = 0.424], or BI score [*t*(232) = 0.24, *p* = 0.812].

#### Pilot Survey Measures

The pilot survey consisted of the following measures in order to develop ENACT:

• Evaluating Nature Activities for Connection Tool pilot items: Developed in Phase 1 to assess nature activity aspects that may predict nature-related behavioral intention (20 items; see Table 1)
• Behavioral Intention (BI) index: Developed in Phase 1 (8 items), plus 1 qualitative item asking participants who intended to take action to help nature what action(s) they intended to take (see Supplementary Material 4)
• NR-6 (Nisbet and Zelenski, 2013) to measure trait NC (6 items)
• Connectedness to Nature Scale state version (Mayer et al., 2009) to measure state NC (13 items)
• Demographics (gender, age, highest education level attained; 3 items)

For future analyses of ENACT in relation to different activities, we also recorded the following activity metadata that are not reported here: activity type, reserve, region, cost, weather, date, target audience, and facilitation type.

#### Follow-Up Survey Measures

We conducted the follow-up survey 1 month after each activity. This was a short, online survey to determine, through self-report, the number of nature-related behaviors completed since the activity. It used the same eight nature-related behaviors that were included in the pilot survey BI index but the wording was adjusted to reflect behavior rather than intention. Participants were asked whether or not they had performed each of the eight behaviors since attending the original nature activity (*Yes*, *No*, or *Don’t know*). The participant’s behavior score was the proportion of *Yes* responses (ranging from 0 to 1). To gain a better understanding of the kinds of short-term actions taken, participants who said they had taken action to help nature were then asked whether or not they had taken seven specific types of action, which were chosen based on frequent responses to the pilot survey qualitative item (see Supplementary Material 5 for follow-up survey content).

#### Procedure

We pre-piloted the pilot survey with four participants to check comprehension and completion time, resulting in minor amendments to some item wording. We conducted the pilot survey from August-November 2018 at 19 activities held at 11 RSPB nature reserves in England. We included a wide range of nature activities; from exclusive nature safaris and specialist, guided walks to large-scale drop-in family events and sporting activities. We recruited all survey participants face-to-face, directly post-event and invited them to participate in a 10-min survey about their experience. One author (VC) conducted surveys at 17 activities, both authors covered one activity and a trained colleague covered one activity when we were not available. At drop-in activities where people arrived and left throughout the day, as far as feasible we approached all adults leaving after at least 1 h. At activities where a group started and finished at the same time (e.g., guided walks), where possible we, or an event facilitator, mentioned the research at the start to encourage participation. There were three versions of the survey, which showed the 20 activity aspects in a different random order, to limit potential order effects. Versions were cycled to ensure completion of a roughly even number of each. All surveys were completed on paper for convenience (Robinson, 2018). The survey was either completed verbally, with us recording participants’ responses, or completed independently by the participant (e.g., when two or more participants needed to complete surveys concurrently) while we remained nearby to answer any queries.

Participants were asked to provide their email address if they were willing to participate in a short follow-up survey within 3 months; no further details were provided about the content or timing of the follow-up survey to avoid cueing. Participants who agreed to the follow-up were emailed a unique link to the online survey (SurveyMonkey) exactly 1 month after the activity. The survey included introductory information to enable informed consent and debrief information. It was not possible to complete the survey more than once from the same device. Participants who had not yet completed the follow-up survey received one reminder email after 2 weeks.

#### Analytic Strategy

The Evaluating Nature Activities for Connection Tool aims to assess how effective nature activities are in terms of increasing nature-related behavioral intention and short-term behavior. Therefore, the goal is an applied, evaluative tool for prediction of specific outcomes rather than a comprehensive psychometric measure of a construct (e.g., NC). While we do not see ENACT as a psychometric scale, we have used elements of best practice scale development (e.g., DeVellis, 2017) where appropriate and feasible, in order to make ENACT as robust as possible. Although writing in a clinical context, Smits et al. (2018) described how item selection procedures may differ from typical scale development procedures when designing a tool to predict a specific outcome. Tools for prediction prioritize correlation with the outcome measure and may result in lower inter-item correlations and reliability, whereas typical procedures that prioritize factor analysis and scale reliability may result in sub-optimal tools for prediction. As ENACT is a predictive tool and we wanted to select the best subset of predictors from the pilot items, item selection was based on correlation and regression to predict the outcome measure, with reliability and factor analysis used later to evaluate the model.

Cross-validation is recommended when using multiple regression to create a model, particularly when stepwise regression is used (Churchill, 1979; Follows and Jobber, 2000; Tabachnick and Fidell, 2001; Field, 2009). To cross-validate the item selection, the pilot data (*N* = 241) was split into a training sample (*N* = 193) and a testing sample (*N* = 48) using a randomized 80/20 split (Tabachnick and Fidell, 2001). The training sample size was above the power analysis recommended sample size for *R*^2^ = 0.20 (*N* = 101). As the aim of the regression was to create a prediction equation that identified a subset of factors useful in predicting the outcome, and eliminate predictors that did not add predictive value, stepwise regression was appropriate (Tabachnick and Fidell, 2001). It was not possible to theoretically deduce the order of importance of predictors in order to conduct a hierarchical regression and, due to inter-item correlations, standard forced-entry multiple regression may not have identified the subset of predictors that together best predict the outcome (Tabachnick and Fidell, 2001). Stepwise regression has been found to be as robust as other methods for predictor selection using multiple regression (Murtaugh, 2009). All items were included as suppression effects mean lower zero-order correlations may change when the items are included in a larger regression model where other items are controlled for (MacKinnon et al., 2000; Korpela et al., 2008). To avoid overfitting, the more conservative forward stepwise regression is recommended over backward when there is a large number of possible predictors and has been used in previous research with a similar aim (e.g., Korpela et al., 2008). A liberal criterion where the minimum probability of F is 0.15 to 0.20 is recommended (rather than 0.05), for entry of predictors into a forward regression so that important variables are less likely to be excluded from the model (Bendel and Afifi, 1977; Tabachnick and Fidell, 2001). Furthermore, sufficient predictors are needed to create a reliable tool covering a range of activity aspects; some researchers have recommended a minimum tool length of eight items (Carifio and Perla, 2007).

To explore the validity of ENACT, including relationships with state and trait NC, demographics, and reported behavior at follow-up, we used regression models and correlation analyses on the full data set. We conducted analyses using IBM SPSS Statistics Version 22 (IBM Corp., 2013) and statistical software R (version 3.5.1, R Core Team, 2018), with packages car (Fox and Weisberg, 2019), and mlogit (Croissant, 2019).

### Results

#### Behavioral Intention and Follow-Up Behavior

Using the full data set (*N* = 241), we calculated BI score for participants who responded to all eight items. The BI index showed good internal reliability (α = 0.74) and principal components factor analysis suggested a single factor. Although the three “repeating nature activities” items could be interpreted as a separate factor, this subset was not sufficiently reliable to be used in isolation. The behaviors that participants most often “probably” or “definitely” intended to do in the next month were: tell other people about the event (86%), visit a different nature reserve (78%), and spend more time in natural places (71%). 59% (*N* = 141) of participants probably/definitely intended to take action to help nature; 111 of these participants specified actions they intended to take. The most frequent intended actions were: feeding birds or other wildlife, recycling or reducing waste, volunteering, nature-friendly gardening, and eco-friendly consumption (e.g., purchasing choices). At follow-up (*N* = 145), the behaviors that participants most often said they had done since the event were: told other people about the event (90%), spent more time in natural places (59%), and taken action to help nature (55%). Of those who had taken action to help nature (*N* = 80), the actions reported most frequently were feeding birds or other wildlife in my garden or local area (86%) and recycling more or reducing waste (73%); all other types of action were taken by less than half of these participants.

#### ENACT Item Selection

Using the training sample (*N* = 193), we examined the zero-order correlations between the 20 activity aspects and BI score, using Spearman’s rank correlations due to ordinal single-item data and some skewed item distributions. Table 1 shows means, standard deviations and correlations with BI for the 20 activity aspects. All items showed significant positive correlations with BI (*p* ≤ 0.008); the highest correlations were the two Emotion items [*r*_s_(184) = 0.45 and *r*_s_(183) = 0.47], Learning [*r*_s_(182) = 0.47], and Stress Distraction [*r*_s_(182) = 0.46]. There were significant positive inter-correlations between the majority of items, the highest being *r*_s_(187) = 0.72 and *r*_s_(188) = 0.70 [below Field’s (2009) suggested threshold of *r* = 0.80 for multicollinearity]; all other inter-item correlations were r_s_ ≤ 0.57, with a mean inter-item correlation of *r*_s_ = 0.33. As all aspects correlated with the outcome and most were inter-correlated, regression was critical to determine the best item selection. We conducted a forward stepwise regression with all 20 items included as potential predictors. When the minimum *F* probability criterion was set at 0.15, forward regression selected eight items and at 0.20 selected 11 items. Graphical analysis of the curve of diminishing gains in *R*^2^ with increasing predictors suggested that the curve plateaued at 11 items. Furthermore, one of the items selected between 8th and 11th showed greater increase in *R*^2^ than an item selected earlier, suggesting a benefit in using a criterion of 0.20. The 11-item selection included the four items with the highest zero-order correlations, giving confidence that potentially important variables were included. Table 2 shows the output of the final regression, with the 11 items of ENACT (see Supplementary Material 6 for ENACT content).

**TABLE 2:** Predictors of behavioral intention with 20 activity aspects entered as initial independent variables, statistical regression analysis (forward with PIN of 0.20; *N* = 158).

	*R*^2^	Adj *R*^2^	Unstandardized coefficients		Standardized beta	*t*	Sig.	95% CI for B		Correlations		Collinearity statistics
			B	Std error				Lower bound	Upper bound	Zero-order	Partial	Tolerance
(Constant)			2.11	0.39		5.48	0.000	1.35	2.87			
Learning: *I learned something new about nature*	0.24	0.23	0.15	0.04	0.35	4.24	0.000	0.08	0.23	0.49	0.33	0.55
Stress Distraction: *It took my mind off stresses or problems*	0.34	0.33	0.13	0.05	0.21	2.69	0.008	0.03	0.22	0.47	0.22	0.61
Facilitation: *The staff/volunteers were knowledgeable*	0.37	0.36	0.18	0.09	0.17	2.03	0.045	0.00	0.36	0.38	0.17	0.53
Meaning: *This place means something to me*	0.38	0.37	0.08	0.04	0.14	2.06	0.041	0.00	0.16	0.35	0.17	0.78
Interest: *It was interesting and informative*	0.40	0.38	−0.08	0.06	−0.12	−1.44	0.151	−0.19	0.03	0.29	−0.12	0.52
Contact: *I used different senses to experience nature (sight, sound, smell, touch)*	0.41	0.38	0.08	0.04	0.15	2.08	0.039	0.00	0.16	0.35	0.17	0.70
Non-disturbance: *I was able to enjoy wildlife without disturbing it*	0.42	0.39	−0.10	0.05	−0.17	−2.19	0.030	−0.19	−0.01	0.19	−0.18	0.62
Emotion: *It made me feel calm and relaxed*	0.43	0.40	0.10	0.06	0.13	1.67	0.098	−0.02	0.22	0.41	0.14	0.57
Emotion*: It made me feel excited and amazed*	0.44	0.40	0.10	0.05	0.17	1.97	0.050	0.00	0.19	0.44	0.16	0.52
Organization: *It was well organized*	0.45	0.41	−0.13	0.07	−0.14	−1.76	0.080	−0.27	0.02	0.17	−0.14	0.63
Compassion: *It made me more concerned about the problems facing nature*	0.46	0.41	−0.06	0.04	−0.11	−1.44	0.151	−0.14	0.02	0.17	−0.12	0.68

Collinearity diagnostics did not reveal any problems of multi-collinearity. Assumptions of normality, linearity and homoscedasticity of residuals, and independence of errors, were met (Tabachnick and Fidell, 2001; Field, 2009). There was little evidence of problematic outliers; three cases had a Mahalanobis distance above critical value, but omitting these cases produced the same regression result so they were retained (Field, 2009). Adjusted *R*^2^ was similar to *R*^2^ (slight shrinkage from *R*^2^ = 0.46 to Adj. *R*^2^ = 0.41), a positive indication for model generalizability beyond the current sample (Field, 2009).

For participants who completed all 11 items, we calculated ENACT score as the mean of the 11 selected items. Mean scores are acceptable for most exploratory research situations and were used rather than factor scores or scores derived from regression weights because they are more readily interpretable and facilitate easier use of ENACT in future (Tabachnick and Fidell, 2001; DiStefano et al., 2009). As ENACT score will be used in future research to assess the effectiveness of nature activities, we calculated a simple linear regression to predict BI based on ENACT score alone. ENACT score predicted 31% of the variance in BI [*R*^2^ = 0.31, *F*(1,166) = 73.66, and *p* < 0.001]. The regression equation was BI = 1.705 + (0.545 × ENACT).

#### Cross Validation

To cross-validate the item selection, we used the ENACT score regression equation to predict BI scores in the testing sample (*N* = 48; Tabachnick and Fidell, 2001). The correlation between predicted and actual BI scores was *r*(43) = 0.54, giving an *R*^2^ of 0.29, similar to *R*^2^ = 0.31 observed in the training sample. The training sample equation predicted results in the testing sample reasonably well, providing support for the generalizability of the results. A dependent *t*-test showed that there was no significant difference between the predicted (*M* = 3.80, SE = 0.05) and actual BI scores (*M* = 3.84, SE = 0.10) in the testing sample [*t*(44) = −0.55, *p* = 0.58].

#### ENACT Reliability and Validity

We conducted analyses of the full pilot survey sample (*N* = 241), including the follow-up data (*N* = 145), to explore the reliability and validity of the 11-item ENACT. Table 3 shows descriptive statistics and correlations for the main study variables.

**TABLE 3:** Descriptive statistics and correlations for main study variables.

	*N*	Mean	SD	α	ENACT	NR-6	CNS	BI
ENACT	218	3.91	0.68	0.83				
NR-6 (Trait NC)	234	4.21	0.61	0.78	0.30***			
CNS (State NC)	233	5.29	0.93	0.89	0.41***	0.51***		
Behavioral Intention (BI)	234	3.82	0.68	0.74	0.55***	0.38***	0.34***	
Follow up Behaviors±	142	0.50	0.22	–	0.36***	0.26**	0.33***	0.60***

**p < 0.01 ***p < 0.001 ± Spearman’s rho; all other correlations are Pearson’s *r*.

##### Reliability and factor structure

The Evaluating Nature Activities for Connection Tool showed high internal reliability (α = 0.83; Nunnally, 1978) and scores were normally distributed. Principal components factor analysis suggested a one-factor solution based on the scree plot method (Cattell, 1966; Bryman and Cramer, 2001). Although a two-factor solution was also plausible, it was not readily interpretable. The first factor accounted for 38% of the variance, with an eigenvalue of 4.22. All items loaded on it positively from 0.47 to 0.74, with an average factor loading of 0.61. The next eigenvalue was 1.32, accounting for 12% of variance. Only two items had factor loadings over 0.50 on the second factor (Costello and Osborne, 2005), supporting a one-factor solution.

##### Relationship with NC measures

In this study, the NR-6 and state CNS showed high internal reliability (NR-6 α = 0.78; CNS α = 0.89). ENACT was significantly positively correlated with both measures of NC (*p* < 0.001), indicating that ENACT is tapping into elements of NC and providing evidence of convergent validity (Cohen, 1992). As expected, ENACT correlated more strongly with state NC [CNS *r*(209) = 0.41, modest correlation; Cohen and Holliday, 1982; Bryman and Cramer, 2001] than trait NC [NR-6 *r*(210) = 0.30, low correlation], as the latter is unlikely to change in response to a single activity and was therefore seen as a pre-existing, control variable in this study. The correlation with state NC was large enough to provide evidence of concurrent validity (*r* > 0.40; Kline, 2000), but the level of correlation with both measures suggests ENACT also assesses something different and additional to existing NC measures.

##### Prediction of nature-related behavioral intention

Simple linear regression showed that ENACT score predicted 30% of the variance in BI scores in the full sample [*R*^2^ = 0.30, *F*(1,211) = 92.19, and *p* < 0.001]. The regression equation was BI = 1.72 + (0.54 × ENACT). We used hierarchical multiple regression to examine whether ENACT scores predicted BI after controlling for demographic variables, trait and state NC. Age was not correlated with ENACT score but had a small significant negative correlation with BI [*r*(219) = −0.14, *p* = 0.041] so was included in the regression. Similarly, although there was no gender difference in ENACT scores, females (*M* = 3.91, SD = 0.67) scored significantly higher than males (*M* = 3.69, SD = 0.69) on BI [*t*(230) = 2.45, *p* = 0.015], so gender was also included. Educational level was not related to any of the main study variables so was omitted. Table 4 shows the results of a hierarchical multiple regression to predict BI, with person factors (age, gender, and trait NC) entered at Step 1, state NC entered at Step 2 and ENACT score entered at Step 3. At Step 1, person factors explained 19% of the variance in BI (*p* < 0.001), with age and trait NC significant independent predictors. At Step 2, state NC explained an additional 3% of the variance in BI (*p* = 0.007), suggesting that it offers incremental validity over trait NC in predicting BI. At Step 3, ENACT score explained an additional 17% of the variance in BI (*p* < 0.001), showing that ENACT provides incremental validity over existing NC measures in predicting BI. Assumptions regarding normality, linearity and homoscedasticity of residuals, independence of errors and lack of multi-collinearity were met. Adjusted *R*^2^ was very similar to *R*^2^ (*R*^2^ = 0.39, Adj *R*^2^ = 0.38), a positive indication for model generalizability beyond the current sample (Field, 2009).

**TABLE 4:** Hierarchical regression analysis for predicting behavioral intention (BI; *N* = 195).

	*R*^2^	Adj. *R*^2^	*R*^2^Δ	*F* Δ	*B*	SE B	95% CI	β	*t*	*P*
Step 1	0.19	0.18	0.19	14.79***						
Age					−0.01	0.00	[−0.02,0.00]	−0.19	−2.91	0.004
Gender					−0.18	0.09	[−0.36,0.00]	−0.13	−1.94	0.054
NR-6 (trait NC)					0.42	0.08	[0.27,0.57]	0.37	5.51	<0.001
Step 2	0.22	0.20	0.03	7.42**						
CNS (state NC)					0.16	0.06	[0.04,0.27]	0.20	2.72	0.007
Step 3	0.39	0.38	0.17	53.42***						
ENACT					0.46	0.06	[0.33,0.58]	0.46	7.31	<0.001

**p < 0.01, and ***p < 0.001; CI = Confidence Interval for *B*.

##### Prediction of self-reported nature-related behaviors after 1 month

We used logistic regressions (binomial GLMs for proportional data; Thomas et al., 2017) to predict the proportion of self-reported nature-related behaviors completed after 1 month. A simple logistic regression showed that BI was a significant predictor of behavior (Hosmer–Lemeshow *R*^2^ = 0.31, *b* = 0.90, *z* = 8.50, and *p* < 0.001), further supporting the link between behavioral intention and behavior (Ajzen, 1991; Bamberg and Möser, 2007). The significant positive correlation between ENACT score and behavior after 1 month [*r*_s_(130) = 0.37, *p* < 0.001] demonstrates reasonable predictive validity (Cohen, 1992; Robinson, 2018). A simple logistic regression showed that ENACT score was a significant predictor of behavior (H-L *R*^2^ = 0.14, *b* = 0.55, *z* = 5.61, and *p* < 0.001), providing evidence of the predictive validity of ENACT. We then used a series of logistic regressions to examine whether ENACT scores predicted behavior after controlling for demographic variables, trait and state NC. Females (Mdn = 0.50) reported performing a greater proportion of the behaviors than males (Mdn = 0.38; *U* = 1893.5, *z* = −2.06, *p* = 0.040), so gender was included in the regressions. Age did not correlate with behavior but was included to maintain consistency with the BI regressions. As before, educational level was not related to the main study variables so was omitted. Model 1, with age, gender and trait NC as predictors, explained 10% of the variance in behavior (H–L *R*^2^ = 0.10, *p* < 0.001), with trait NC the only significant predictor (*b* = 0.49, *z* = 4.43, and *p* < 0.001). Model 2, with state NC added, produced a significant improvement in the fit of the model [*x*^2^(2) = 15.79, *p* < 0.001, and H–L *R*^2^ = 0.16] and state NC was the only significant predictor of behavior (*b* = 0.31, *z* = 3.90, and *p* < 0.001). Model 3, with ENACT added, produced a further significant improvement in the fit of the model [*x*^2^(13) = 27.00, *p* = 0.012, and H–L *R*^2^ = 0.19]. ENACT was the only significant predictor of behavior (*b* = 0.42, *z* = 3.63, and *p* < 0.001) in Model 3, showing that ENACT provides some incremental validity over existing NC measures in predicting self-reported behavior after 1 month. Table 5 shows the results of logistic regression Model 3. Data assumptions were checked and met for each model.

**TABLE 5:** Logistic regression model 3 for predicting behavior after 1 month (*N* = 125).

	*b*	SE *b*	*Z*	*P*
Constant	−3.14	0.64	−4.87	<0.001
Age	−0.00	0.00	−0.15	0.878
Gender	−0.16	0.14	−1.13	0.258
NR-6 (trait NC)	0.17	0.14	1.23	0.219
CNS (state NC)	0.17	0.09	1.86	0.063
ENACT	0.42	0.12	3.63	<0.001

*R^2^ = 0.19 (Hosmer–Lemeshow), 0.31 (Cox-Snell), and 0.36 (Nagelkerke).*

Overall, this pattern of findings suggests that ENACT is a reliable tool, shows convergent validity with state NC but also assesses something different to trait and state NC. ENACT shows predictive validity in predicting self-reported nature-related behaviors after 1 month and was a better predictor of behavior than trait and state NC (incremental validity).

## Discussion

Developing people’s NC in order to motivate conservation behavior, has become an important focus for organizations and groups that understand conservation action requires more than just information provision (Toomey et al., 2017). For a variety of ethical, financial and logistical reasons, evaluating the success of interventions is critical; however, this is impossible without the correct tools. Through this study, we have identified aspects common to different nature activities that predict NC, nature-related behavioral intentions and self-reported nature-related behaviors 1 month later. Furthermore, we have designed and validated a usable, simple survey tool, ENACT, that can be used to assess the effectiveness of nature activities which aim to promote adult NC in order to encourage future conservation behavior. ENACT provides key insights into aspects of nature activities that may be crucial for developing or strengthening the type of NC that leads to plausible nature-related behavior outcomes. By focusing on the 11 ENACT aspects within nature activities, it may be possible to maximize NC and increase desired nature-related behavior outcomes.

Nature connection is a broad psychological construct that is linked to a variety of outcomes, from human health and wellbeing to conservation behavior (Capaldi et al., 2014; Mackay and Schmitt, 2019; Whitburn et al., 2019; Martin et al., 2020). However, it may be that different components within NC are more relevant to some outcomes than to others. Attempts to identify specific components of NC that link more strongly with conservation behavior are only just beginning (Hatty et al., 2020). Given an interest in a specific outcome, it is important to identify those aspects of NC that are most strongly predictive of that outcome, and the pathway for developing these. Our analyses demonstrated that ENACT assesses NC to some extent, as it correlated significantly with existing measures of state and trait NC. The aspects of nature activities selected for ENACT reflect previously determined pathways for developing NC, such as contact, emotion, meaning and compassion (Lumber et al., 2017). Crucially, ENACT provided additional information over current NC measures about aspects of nature activities that are related to participants’ ensuing nature-related behavioral intentions and nature-related behavior. It shows that desired nature-related behavior outcomes were more likely among adults that experienced compassion, stress distraction, meaning, interest, contact, relaxing and/or exciting emotions, and learned something new at activities that were well-organized, facilitated by knowledgeable staff and enabled experiencing wildlife without disturbance.

There are some design and contextual limitations to this study that may influence the wider applicability of ENACT. Firstly, there is currently no defined criterion measure for short-term conservation behavior outcomes, which makes prediction challenging (Smits et al., 2018). While approaches such as Community-Based Social Marketing advocate identifying specific target behaviors for interventions (McKenzie-Mohr, 2011), this is not feasible when trying to evaluate effectiveness across a wide range of different activities. We therefore needed to develop a new outcome index as part of this study, in addition to developing ENACT itself. We did this through research with stakeholders at a single, large conservation organization (RSPB), which we assumed to be reasonably representative of the United Kingdom conservation sector, and members of the public attending events at RSPB reserves. While we consider that the resulting BI index comprises generally desirable nature-related behaviors following single-event nature activities, rather than being specific to the RSPB, different groups and organizations may have other desired outcomes that are not fully met by the BI index, and ENACT may not as successfully predict these; further research is planned to explore this. Secondly, as ENACT was developed on activities taking place on nature reserves, a high nature environment, the response to activities may be influenced by the surroundings (Davis and Gatersleben, 2014). It is plausible that ENACT may not be as applicable to nature activities conducted in low nature settings and further research is planned to explore this. In addition, we acknowledge that nature reserve visitors are not representative of the United Kingdom general population, so results may not generalize to other contexts where participants are more typical of the general population. Thirdly, considerations of length in the pilot survey meant that some activity aspects expressed as potentially influential in Phase 1 had to be omitted from the pilot. While the rationale for including only 20 aspects is justifiable, the exclusions mean that other aspects may have been overlooked.

The Evaluating Nature Activities for Connection Tool is, as far as we are aware, one of the only tools that aims to predict likelihood of particular outcomes from efforts to connect people to nature. ENACT provides a useful tool for evaluating the effectiveness of nature activities that have costs to organizations and groups working with limited resources. From this research, practitioners can focus on particular aspects of NC activities in order to promote NC, nature-related behavioral intention and motivate nature-related behavior in the short term. Furthermore, it will be possible to look at comparative efficacy of different types of nature activity in developing NC and improve program evaluation. Experimental research is required to examine how powerful ENACT is. For example, this study raises questions on how to improve ENACT scores through targeted improvements to activities, whether varying BI and behavior items alters the predictive ability of ENACT, and whether ENACT predicts observed behavior as well as self-reported behavior. There are also broader theoretical NC challenges to face which ENACT will contribute toward. These include: identifying how the nature of trait NC differs if specific outcomes are desired (Zylstra et al., 2014) and developing trait NC measures that predict specific outcomes; whether and how changes in state NC translate into long-term changes in trait NC, as widely hypothesized; and how this in turn relates to changes in conservation behavior – for example, in frequency or breadth.

## Conclusion

This study has provided a tool for the evaluation of nature activities aimed at developing adult NC in order to motivate conservation behavior and ultimately increase the likelihood of conservation success. To date, there has been no way of measuring the effect of participating in these activities on people’s intentions to behave more positively for nature, resulting in a plethora of activities with little assessment of their effectiveness in terms of NC. We are confident that ENACT can help the conservation community by enabling organizations and groups to refine and improve nature activities to increase biodiversity conservation success.

## Data Availability Statement

The raw data supporting the conclusions of this article will be made available by the authors, without undue reservation.

## Ethics Statement

The studies involving human participants were reviewed and approved by RSPB Centre for Conservation Science Human Ethics Committee. The participants provided their written informed consent to participate in this study.

## Author Contributions

Both authors contributed to the study conception and design. VC prepared the materials, collected the data, and analyzed the data, with guidance from JH. Both authors wrote the first draft of the manuscript, revised the manuscript, and read and approved the submitted version.

## Conflict of Interest

The authors declare that the research was conducted in the absence of any commercial or financial relationships that could be construed as a potential conflict of interest.
