# Supplementary figures and images for: Predicting the Development of Adult Nature Connection Through Nature Activities: Developing the Evaluating Nature Activities for Connection Tool
Source: Front Psychol. 2021 Mar 23;12:618283. doi: 10.3389/fpsyg.2021.618283 (PMC8044968; doi:10.3389/fpsyg.2021.618283)

**Supplementary Material S1:** Hypothesized Connection Pathway

**
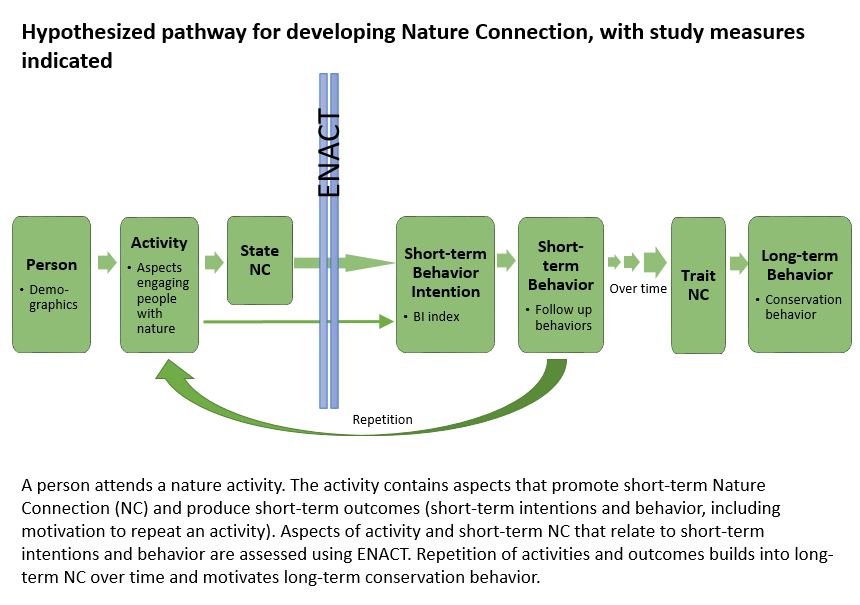
**

Supplement: Supplementary file 1 [file Data_Sheet_1.docx]
