# Supplementary material for: Predicting the Development of Adult Nature Connection Through Nature Activities: Developing the Evaluating Nature Activities for Connection Tool
Source: Front Psychol. 2021 Mar 23;12:618283. doi: 10.3389/fpsyg.2021.618283 (PMC8044968; doi:10.3389/fpsyg.2021.618283)
Supplement: Supplementary file 2 [file Data_Sheet_2.docx]

**Supplementary Material S2:** Nature Activity Participant Interview Schedule

| ID: |  | Event: |  | Date: |  |
| --- | --- | --- | --- | --- | --- |

Just a couple of quick background questions to start:

| 1. How long have you spent on the reserve today? | ________ hours | |
| --- | --- | --- |
| 2. How often do you visit an RSPB reserve? | This is my first time |  |
|  | Once a year or less often |  |
|  | Once every few months |  |
|  | Monthly |  |
|  | Weekly or more often |  |

3. How did this event make you feel?

- Probe: What sorts of things did you feel or think, during or after the event?
- *Alternative if needed: What was the best thing about this event? Why?*

4. What was it about this event that made you feel like that?

- Probe: Was there something that happened, or something that someone did, that made you feel that way – why? At what point did you most feel like that – why?

5. What did you want to get out of this event and did you achieve this?

- If YES: Was there anything in particular that helped you achieve this?
- If NO: What would have helped you to achieve this?
- *Alternative if needed: How would you have completed this sentence beforehand: This event will be good or worthwhile if…..*

6. Did this event help you to feel more connected to nature?

- *Alternative if needed: …feel closer to nature OR feel part of the natural world?*
- If YES: In what way? What does that feel like? What was it about the event that made you feel more connected/closer to nature?
- If NO: Why not? What would have made you feel more connected/closer to nature?

7. Following this event, in what ways (if any) do you feel, think or plan to act differently?

- Probe: Do you plan to do anything as a result, e.g. visit/do something similar again or tell others about your experience? Why / why not? What does this depend upon?

Just a couple of questions about you to finish. We use these just to give summary details about the group of people we have interviewed.

| 8. What is your gender or gender identity? | Female |  |
| --- | --- | --- |
|  | Male |  |
|  | Non binary/another gender identity |  |
| 9. What is your age? | ________ years | |
