# Supplementary material for: Predicting the Development of Adult Nature Connection Through Nature Activities: Developing the Evaluating Nature Activities for Connection Tool
Source: Front Psychol. 2021 Mar 23;12:618283. doi: 10.3389/fpsyg.2021.618283 (PMC8044968; doi:10.3389/fpsyg.2021.618283)
Supplement: Supplementary file 3 [file Data_Sheet_3.docx]

**Supplementary Material S3:** Phase 1 Qualitative Themes

**Table 1:** Nature activity outcomes themes

| **Activity outcomes** | **% Interviews mentioning** | **Focus group** | **BI index item** |
| --- | --- | --- | --- |
| Return to this nature reserve | 83% | Yes | I intend to visit this nature reserve again |
| Tell others/recommend | 47% | Yes | I intend to tell other people about today’s event |
| Visit other reserves | 33% | Yes | I intend to visit a different nature reserve |
| Take action for nature/environment | 27% | Yes | I intend to take action to help nature |
| Share on social media | 20% | Yes | I intend to share photographs or information from today’s event on social media |
| Seek further information relevant to today | 20% | Yes | I intend to look for further information relating to something I have done or seen today |
| Other follow up activities, e.g. art | 20% | No |  |
| Visit/spend time in natural places | 17% | No | I intend to spend more time in natural places |
| Join RSPB | 13% | No |  |
| Encourage others into nature/action | 13% | No |  |
| Find out how to help/support nature | 7% | Yes | I intend to find out what I can do to help or support nature |
| Volunteer | 7% | No |  |
| Take physical activity | 7% | No |  |
| Barriers to behavior, e.g. time | 7% | No |  |

**Table 2:** Nature activity aspects themes

| **Activity aspects** | **% Interviews mentioning** | **Focus group** | **Literature search reference** | **ENACT pilot item(s)** |
| --- | --- | --- | --- | --- |
| Contact | 70% | Yes | Lumber et al., 2017 | I got up close to nature;  I used different senses to experience nature (sight, sound, smell, touch) |
| Beauty | 53% | No | Lumber et al., 2017 | I took time to appreciate my surroundings;  I noticed beautiful things in nature |
| Emotion (general) | 10% | Yes | Lumber et al., 2017 | It made me feel calm and relaxed;  It made me feel excited and amazed |
| Meaning | 33% | Yes | Lumber et al., 2017 | This place means something to me |
| Compassion | 10% | Yes | Lumber et al., 2017 | It made me more concerned about the problems facing nature;  It made me feel more responsible for protecting nature |
| See something new/special | 83% | Yes |  | I saw wildlife/nature that I had never, or hardly ever, seen before |
| Enjoyment (general) | 73% | Yes | Natural England, 2018 | Global item - I enjoyed it |
| Facilitator knowledgeable/passionate | 63% | Yes |  | The staff/volunteers were knowledgeable |
| Awe/wonder/excitement | 60% | Yes | Perkins, 2010; Yang et al., 2018 | It made me feel excited and amazed |
| See named/target species | 57% | No |  | I saw wildlife/nature that I had never, or hardly ever, seen before |
| Being outdoors/fresh air | 57% | No |  | n/a - presumed present in all activities |
| Calm/peaceful/relaxed | 50% | No | Natural England, 2018 | It made me feel calm and relaxed |
| Surroundings (specific) | 50% | No | Natural England, 2018 | I took time to appreciate my surroundings |
| Interesting/engaging/stimulating | 47% | Yes |  | It was interesting and informative |
| Privileged/special access | 43% | No |  | I had privileged access to natural places |
| Shared/social experience with others | 43% | Yes | Mayer et al., 2009 | I saw that other people were interested in nature |
| Weather | 40% | No |  | n/a - collect at event level |
| See nature conserved/conservation effort | 40% | Yes |  | It made me aware of the conservation work being done here |
| Satisfied/exceeded expectations | 40% | Yes |  | It exceeded my expectations |
| Wellbeing/de-stressing/health | 37% | Yes | Korpela et al., 2008 | It took my mind off stresses or problems |
| Amount/variety of wildlife/nature | 33% | No |  | I felt surrounded by nature |
| Learning/understanding | 33% | Yes | Lumber et al., 2017; Natural England, 2018 | I learned something new about nature |
| Sense/meaning of place | 33% | Yes | Masterson et al., 2017 | This place means something to me |
| Oneness/place in world | 30% | Yes | Mayer et al., 2009 | n/a - measured by state NC |
| Busyness of event | 30% | No |  | I saw that other people were interested in nature |
| Surroundings (general) | 30% | No | Natural England, 2018 | I took time to appreciate my surroundings |
| Appreciation/conscious engagement | 30% | Yes | Natural England, 2018 | I took time to appreciate my surroundings |
| See wildlife naturally/no interference | 27% | No |  | I was able to enjoy wildlife without disturbing it |
| Organisation of event/venue | 27% | Yes |  | It was well organised |
| Involve children/see them interested | 27% | Yes |  | I saw that other people were interested in nature |
| Threat/loss of nature/concern | 27% | Yes |  | It made me more concerned about the problems facing nature |
| Accessibility/proximity of event | 23% | Yes |  | n/a |
| Anticipation | 20% | No |  | n/a |
| Responsibility/guilt | 20% | Yes | Bamberg and Möser, 2007 | It made me feel more responsible for protecting nature |
| Intensity/immersion in nature | 17% | Yes |  | I felt surrounded by nature |
| Achieved goal/needs | 17% | Yes |  | n/a |
| Personal benefit | 17% | Yes |  | n/a |
| Physicality/active participation | 13% | Yes |  | n/a |
| Impressed | 13% | No |  | n/a |
| Inspired | 13% | No |  | n/a |
| Ambience/atmosphere | 13% | No |  | n/a |
| Empathy/sympathy | 10% | No | Cheng & Monroe, 2012 | n/a |
| Ease of taking action/self-efficacy | 10% | Yes | Ajzen, 1991; Bandura, 1982^1^ | n/a |
| Escape/freedom | 10% | No |  | n/a |
| Nostalgia | 10% | Yes |  | n/a |
| Creating memories | 10% | Yes |  | n/a |
| Self-reflection | 7% | No | Richardson & Sheffield, 2015 | n/a |
| Fun/humour/joy | 7% | Yes |  | n/a |
| Relevance | 3% | Yes |  | n/a |
| Decreased barriers to nature | 3% | Yes |  | n/a |
| Awareness | 3% | Yes |  | n/a |
| Refreshed/revitalised | 3% | No | Natural England, 2018 | n/a |
| Anthropomorphism | 3% | No | Tam et al., 2013^2^ | n/a |

^1^ Bandura, A. (1982). Self-efficacy mechanism in human agency. Am. Psychol. 37, 122–147. doi: 10.1037/0003-066X.37.2.122

^2^ Tam, K.-P., Lee, S.-L., and Chao, M. M. (2013). Saving Mr. Nature: anthropomorphism enhances connectedness to and protectiveness toward nature. J. Exp. Soc. Psychol. 49, 514–521. doi: 10.1016/j.jesp.2013.02.001
