# Supplementary material for: Predicting the Development of Adult Nature Connection Through Nature Activities: Developing the Evaluating Nature Activities for Connection Tool
Source: Front Psychol. 2021 Mar 23;12:618283. doi: 10.3389/fpsyg.2021.618283 (PMC8044968; doi:10.3389/fpsyg.2021.618283)
Supplement: Supplementary file 4 [file Data_Sheet_4.docx]

**Supplementary Material S4:** Behavioral Intention (BI) Index

These statements are about your intentions for the future. Please rate the extent to which you agree with each statement, using the scale shown below.

| Definitely not  (1) | Probably not  (2) | Maybe  (3) | Probably  (4) | Definitely  (5) |
| --- | --- | --- | --- | --- |

*Within the next month…*

1. I intend to visit this nature reserve again

2. I intend to visit a different nature reserve

3. I intend to spend more time in natural places

4. I intend to tell other people about today’s event

5. I intend to share photographs or information from today’s event on social media

6. I intend to look for further information relating to something I have done or seen today

7. I intend to find out what I can do to help or support nature

8. I intend to take action to help nature

9. *(if answer Probably/Definitely to Q8)* What action(s) do you intend to take to help nature? *(free text response)*

Notes:

- BI score is calculated as the mean score across items 1-8.
- Item 9 is a qualitative item used for the pilot survey only; it is not part of the BI index.
