# Supplementary material for: Predicting the Development of Adult Nature Connection Through Nature Activities: Developing the Evaluating Nature Activities for Connection Tool
Source: Front Psychol. 2021 Mar 23;12:618283. doi: 10.3389/fpsyg.2021.618283 (PMC8044968; doi:10.3389/fpsyg.2021.618283)
Supplement: Supplementary file 5 [file Data_Sheet_5.docx]

**Supplementary Material S5:** Follow-up Survey Content

This survey is about your experiences **since the RSPB event/activity where you completed the first survey**. There are no right or wrong answers. Please be frank as this will give us the most useful data for our research.

| Since I completed the first survey…. | Yes | No | Don’t know |
| --- | --- | --- | --- |
| 1. I have revisited the nature reserve where I attended the event |  |  |  |
| 2. I have visited a different nature reserve |  |  |  |
| 3. I have spent more time in natural places |  |  |  |
| 4. I have told other people about the event I attended |  |  |  |
| 5. I have shared photographs or information from the event on social media |  |  |  |
| 6. I have looked for further information relating to something I did or saw at the event |  |  |  |
| 7. I have found out what I could do to help or support nature |  |  |  |
| 8. I have taken action to help nature |  |  |  |

9. (*Only show Q9 if answer Yes to Q8, otherwise skip to Q10*)

Please select any actions you have taken to help nature since you completed the first survey (select all that apply).

| I have fed birds or other wildlife in my garden or local area |  |
| --- | --- |
| I have made a home for nature (e.g. bug hotel) or made my garden more wildlife-friendly |  |
| I have volunteered for an organisation that helps nature |  |
| I have become a member of an organisation that helps nature |  |
| I have given money to help nature (not including membership fees) |  |
| I have recycled more or reduced waste |  |
| I have taken action to increase children’s interest in nature |  |
| Other – please specify *(free text)* |  |

| 10. Finally, thinking about the **last month**, how often, on average, have you spent your leisure time out of doors, away from your home? *This could be anything from a few minutes to all day but does not include routine shopping trips or time spent in your own garden.* | More than once per day |  |
| --- | --- | --- |
|  | Every day |  |
|  | Several times a week |  |
|  | Once a week |  |
|  | Once or twice |  |
|  | Never |  |

Thank you so much for your time.
