# Supplementary material for: Predicting the Development of Adult Nature Connection Through Nature Activities: Developing the Evaluating Nature Activities for Connection Tool
Source: Front Psychol. 2021 Mar 23;12:618283. doi: 10.3389/fpsyg.2021.618283 (PMC8044968; doi:10.3389/fpsyg.2021.618283)
Supplement: Supplementary file 6 [file Data_Sheet_6.docx]

**Supplementary Material S6:** Evaluating Nature Activities for Connection Tool (ENACT)

For these statements, please think about today’s event/activity only and tick the box that best describes your view. There are no right or wrong answers; please respond as you really feel.

| Not at all  (1) | A little  (2) | Somewhat  (3) | Quite a bit  (4) | A great deal  (5) |
| --- | --- | --- | --- | --- |

1. I learned something new about nature

2. I used different senses to experience nature (sight, sound, smell, touch)

3. I was able to enjoy wildlife without disturbing it

4. It made me feel calm and relaxed

5. It made me feel excited and amazed

6. It made me more concerned about the problems facing nature

7. It took my mind off stresses or problems

8. It was interesting and informative

9. It was well organized

10. This place means something to me

11. The staff/volunteers were knowledgeable*

Notes:

- *In the final version of ENACT, this item has an additional response option of “N/A – I did not see any” which was not used in the pilot.
- ENACT score is calculated as the mean score across items 1-11.
